# Impaired telomere maintenance in Alazami syndrome patients with LARP7 deficiency

**DOI:** 10.1186/s12864-016-3093-4

**Published:** 2016-10-17

**Authors:** Brody Holohan, Wanil Kim, Tsung-Po Lai, Hirotoshi Hoshiyama, Ning Zhang, Anas M. Alazami, Woodring E. Wright, M. Stephen Meyn, Fowzan S. Alkuraya, Jerry W. Shay

**Affiliations:** 1 Department of Cell Biology, University of Texas Southwestern Medical Center, Dallas, TX 75390 USA; 2 Department of Genetics, King Faisal Specialist Hospital and Research Center, Riyadh, Saudi Arabia; 3 The Hospital for Sick Children, Department of Pediatric and Molecular Genetics, University of Toronto, Toronto, ON M5s1A8 Canada; 4 Department of Anatomy and Cell, College of Medicine, Alfaisal University, Riyadh, Saudi Arabia; 5 Department of Pediatrics, King Khalid University Hospital and College of Medicine, King Saud University, Riyadh, Saudi Arabia; 6 Center for Excellence in Genomics Medicine Research, King Abdulaziz University, Jeddah, Saudi Arabia

## Abstract

**Background:**

Loss of function in genes required for telomere maintenance result in disorders known as telomeropathies, which are characterized by a pattern of symptoms including generalized and specific lymphocytopenias as well as very short telomere length and disease anticipation.

**Methods:**

Because human LARP7 is the most likely ortholog of the *Tetrahymena* p65 protein, which is required for telomerase activity in that organism, we investigated the effects of LARP7 silencing in human cells as well as in two distinct families with Alazami syndrome (loss of function of LARP7).

**Results:**

Depletion of LARP7 caused a reduction in telomerase enzymatic activity and progressively shorter telomeres in human cancer cell lines. Alazami syndrome patients from two separate cohorts exhibited very short lymphocyte telomeres. Further, wild-type offspring of LARP7 mutant individuals also had very short telomeres, comparable to what is observed in telomerase (hTERT) mutant cohorts.

**Conclusions:**

Together, these experiments demonstrate that in addition to the readily apparent developmental disorder associated with LARP7 deficiency, an underlying telomeropathy exists even in unaffected siblings of these individuals.

## Background

Telomeropathies are disorders of impaired telomere maintenance, which are characterized by symptoms which can include short telomeres, anemia, idiopathic pulmonary fibrosis, intrauterine growth retardation and the “classic triad” of keratosis, nail dystrophy and leukoplakia [1]. Mutations in genes required for telomere maintenance, such as the telomerase catalytic and RNA components, TERC and TERT have been found to cause telomeropathies in an autosomal dominant pattern of inheritance; loss-of-function in a number of other genes such as DKC1, NHP2, TCAB1, TIN2/TINF2, RTEL1, PARN, and CTC1 [1, 2] have also been shown to cause telomeropathies through interference with one or more steps required for telomere maintenance. Importantly, a number of the telomeropathies demonstrate a developmental phenotype in a recessive manner characterized by the overt symptoms of telomeropathy, while individuals who are carriers for the mutation are phenotypically normal but still perpetuate the inter-generational decrease in telomere length [3]. In later generations of a family with such a mutation, diseases such as idiopathic pulmonary fibrosis and cirrhotic liver disease can manifest in individuals who carry a mutant allele but display no other symptoms of a telomeropathy [1]. Because of the increasing number of genes that when mutated can induce telomeropathies as well as the very large number of genes known to modulate telomere length in model organisms such as *Saccharomyces cerevisiae* [4–7], it is likely that many telomere maintenance disorders remain undiscovered in humans, and that further exploration of premature telomere length shortening in humans will reveal novel telomeropathies.

La Autoantigen Related Protein 7 (LARP7) is part of a family of proteins that includes LARP3 (previously known as the La antigen). LARP3 has been shown to act as a negative regulator of telomerase activity via binding to TERC, the RNA component of telomerase [8]. Further, LARP7 is the human ortholog of the p65 protein in *Tetrahymena thermophila* that is required for telomerase assembly in that organism [9]. LARP7 knockout mice display a defect in primordial germ cell development driven by an increase in expression of p15^INK4b^ [10]. In addition, embryonic stem cells deficient for LARP7 exhibit decreased levels of Lin28 mRNA, compromising their self-renewal [11]. In *Tetrahymena*, depletion of the LARP7 ortholog, p65, leads to lower levels of TERT protein as well as telomerase RNA (TERC) component abundance, in addition to its role in telomerase holoenzyme assembly [9, 12, 13]. Because of the many associations between the probably ortholog of LARP7 in *Tetrahymena* and telomerase, as well as the known telomerase-relevant function of the La-family protein LARP3, we decided to investigate how LARP7 impacts telomere maintenance in humans, both in tissue culture as well as in two independent cohorts of human LARP7 loss-of-function mutants.

## Methods

A clonal population of HeLa cells (Hela-3) were harvested for western assays via trypsinization (5 min at 37 °C), and then counted with the TC automated cell counter (Bio-Rad). Cells were then pelleted by centrifugation at 700 rpm for 3 min, then washed with cold (pre-chilled on ice) PBS 2×. A final pelleting step at 2000 rpm for 3 min occurred before the cell pellets were resuspended in RIPA buffer (150 mM NaCl, 50 mM Tris-HCl pH 7.4, 1 mM EDTA, 1 % TritonX-100, 1 % sodium deoxycholic acid, 0.1 % SDS) at a ratio of 50uL per one million cells. Cells were lysed through three cycles of 20 s vortex steps and 5 min on ice, then centrifuged at 20,000 rpm for 10 min. Samples were then denatured by incubation at 95 °C for 5 min. Protein concentration was quantitated with the BCA protein assay kit (Pierce) according to the manufacturer’s instructions. Samples were loaded into 10 well preformed Mini Protein TGX gradient polyacrylamide gels (Bio-Rad) and run at 100 V until the lowest ladder band reached the bottom of the gel. Proteins were transferred to a PVDF membrane using a trans-blot turbo pack (Bio-Rad). The membrane was then washed 2× with PBST, then blocked with a 5 % milk in PBST solution for 1 h at room temperature on a shaker. The LARP7 primary antibody (Abcam) was used at a concentration of 1:5000 following blocking and allowed to hybridize overnight at 4 °C. The membrane was then rinsed 3× with PBST at room temperature on a shaker. The anti-rabbit secondary antibody was used at a concentration of 1:5000 in a 5 % milk PBST solution and incubated at room temperature for 1 h on a shaker. After administration of the secondary antibody, the membrane was washed 3× with PBST, and signal was detected with ECL plus western blotting detection reagents (Amersham). Signal was obtained with the G Box imaging system (Syngene).

Terminal Restriction Fragment (TRF) and droplet digital Telomere Repeat Amplification Protocol (ddTRAP) assays were performed as previously described [14, 15]. Analysis of telomerase splicing was performed as described in [16], with the exception that PCR and quantitation were performed using the droplet digital PCR platform (Bio-Rad, Berkeley, CA). Flow-FISH telomere measurement was accomplished by Repeat Diagnostics, Inc (Vancouver, Canada) according to their standard methods.

### Statistical analysis

Signals from the ddTRAP and splicing assays were compared via two-tailed t-tests and were considered significant if *P* < 0.05. Though Universal STELA was performed as previously [17, 18], we developed a new quantification method which streamlines the workflow and reduces user-induced variability in results. Universal Single TElomere Length Analysis (STELA), a ligation and PCR-based method, allows for detection of critically short telomeres by small amount of genomic DNA. However, manual quantification of the length and dynamics of each telomere band from Universal STELA is error prone and time consuming. Thus, it is important to design a program that can effectively and efficiently identify and analyze those images. We developed a Matlab-based algorithm which can automatically detect bands, calculates average telomere size and the ratio of the short telomeres to the rest based on Universal STELA gel images. Images are sharpened and background subtracted to ensure that bands are distinguishable. The algorithm then identifies the centers of all lanes based on the intensity profile, and identifies bands by locating peaks in the intensity profile. The results are consistent with the previous manual quantification method and can be manually adjusted in case there are false positive/negative outcomes. Furthermore, using this algorithm makes high throughput analysis possible and can avoid artificial bias.

## Results

**Fig. 1:**
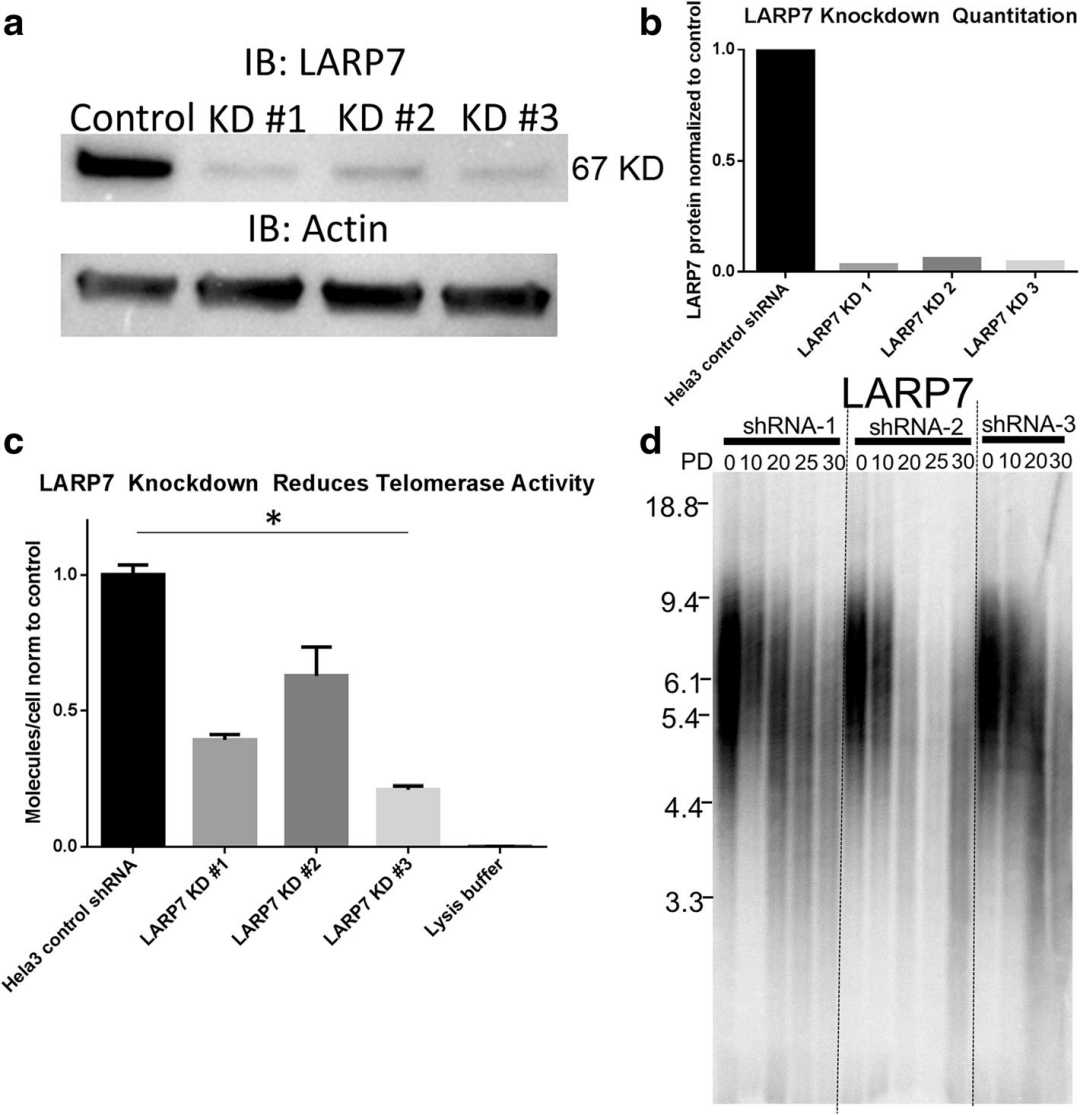
In vitro behavior of LARP7 knockdown cells. Extent of LARP7 knockdown normalized to Actin protein expression in Hela3 cells (**a**) demonstrates ~90 % loss of LARP7 protein in all three shRNAs examined (**b**) (*n* = 1 for each knockdown). Loss of LARP7 is accompanied by reduction in telomerase enzymatic activity (**c**) (*n* = 3) and progressive time-dependent telomere shortening (**d**)

To determine how LARP7 impacts telomere maintenance, we stably knocked down LARP7 with three different shRNA constructs. All three constructs resulted in >90 % reduction in LARP7 protein abundance (Fig. 1a, b). We next evaluated the effect of LARP7 knockdown on telomerase enzymatic activity. All three constructs significantly resulted in a decrease in telomerase enzymatic activity (*p* < 0.01), with a reduction in telomerase enzymatic activity between 40 and 80 % (Fig. 1c). This reduction in telomerase enzymatic activity was accompanied by progressive telomere shortening in all three cell populations upon extended passage in tissue culture (Fig. 1d). These results illustrate that telomerase is not in excess even in cancer cells such that the remaining telomerase enzymatic activity in LARP7 stable knockdown cells was not sufficient to maintain telomere length.

**Fig. 2:**
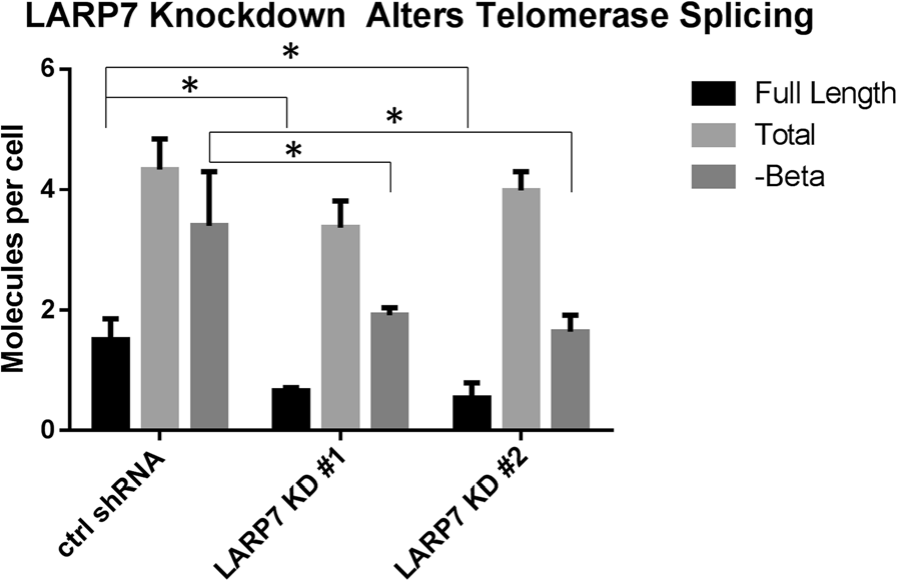
Telomerase splicing in LARP7 knockdown cells. LARP7 knockdown in Hela3 cells leads to statistically significant reductions (*p* < 0.05) in telomerase full-length and -Beta mRNA splicing in both shRNAs evaluated (*n* = 3)

Because LARP7 depletion causes dysregulation of transcriptional elongation as well as aberrant splicing [19], we next evaluated if LARP7 knockdown resulted in changes to the splicing patterns of the telomerase pre-mRNA. LARP7 knockdown caused a significant reduction in full-length telomerase transcripts as well as a reduction in the abundance of the –Beta apparently nonfunctional isoform [20], but did not result in a statistically significant overall reduction in total telomerase mRNA (Fig. 2). This suggests that the reduction in telomerase enzymatic activity was mediated through an effect other than down-regulation of total telomerase transcription.

In contrast to what would be expected based on the observations that LARP7 deficiency in *Tetrahymena* leads to lower levels of TERC RNA and TERT protein [9, 12, 13], in human cells we show that LARP7 stable knockdown does not reduce total telomerase mRNA levels. However, we did observe a reduction in both full-length, functional telomerase mRNA, as well as the non-functional –Beta isoform, indicating a shift toward a splice form other than the two most abundant forms of telomerase mRNA.

In order to determine if LARP7 has the same functions in vivo with regard to telomere maintenance, we performed telomere length analysis in lymphocyte DNA extracted from humans with Alazami syndrome, both the cohort originally described [21] as well as in a Canadian family with a single affected individual (8 years old) and two parents that were carriers for a distinct loss-of-function mutation in LARP7 (a one-nucleotide deletion which causes a frameshift and premature stop, c.756_757del, p.Arg253Ile*6). The Canadian cohort was identified through whole-genome sequencing of the proband, who has long standing growth retardation (< -3.5 SD), moderate developmental delays without regression, axial hypotonia and dysmorphic features (scaphocephalic with frontal bossing/prominent forehead, depressed and wide nasal bridge with a mildly bulbous tip of the nose, telecanthus with mild hypertelorism, prominent ears and small hands with short distal phalanges and nails). The proband has normal brain anatomy (via brain MRI/MRS), small kidneys (*R* < 5%tile and L between 5 and 10 %tile) (via multiple abdominal ultrasounds), several small atrial septal defects and mild left pulmonary artery stenosis, and thin, short distal phalanges of the hands (via skeletal X-ray survey). He also has longstanding borderline anemia (red blood cell counts in the 3.9–4.3 range, with hematocrits in the 34–38 range), but has not been symptomatic for anemia. His white blood cell and platelet counts are normal, and he does not display evidence of the hair, nail or skin abnormalities normally observed in classical Dyskeratosis congenita. Both of his parents carry one copy of the deletion and have no history of anemia, lung disease or mucocutaneous abnormalities.

**Fig. 3:**
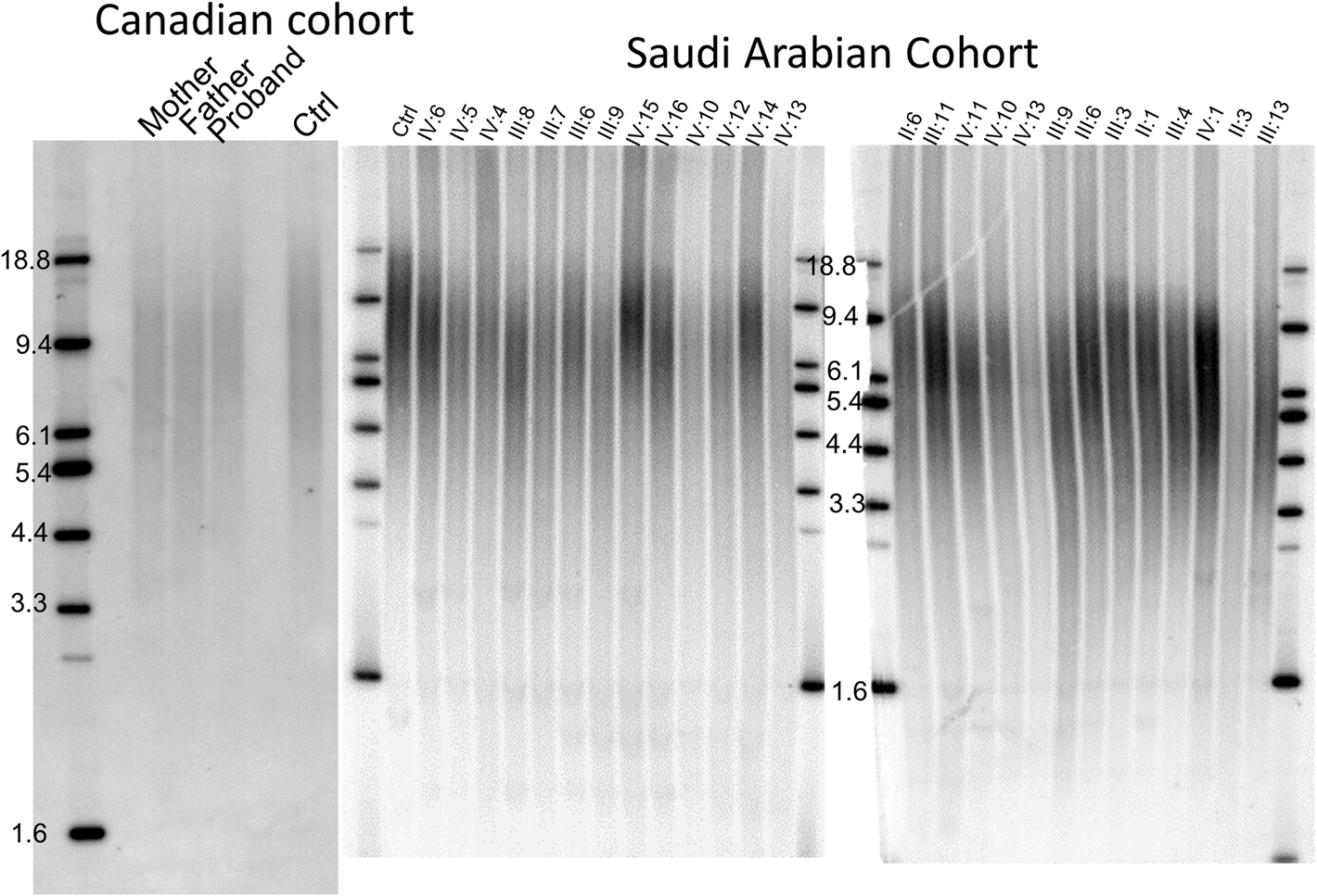
Telomere length measurement of LARP7 mutant cohorts. Terminal Restriction Fragment (TRF) assays for the Saudi and Canadian cohorts illustrate short lymphocyte telomeres, even in individuals who do not manifest the developmental symptoms of Alazami syndrome

**Fig. 4:**
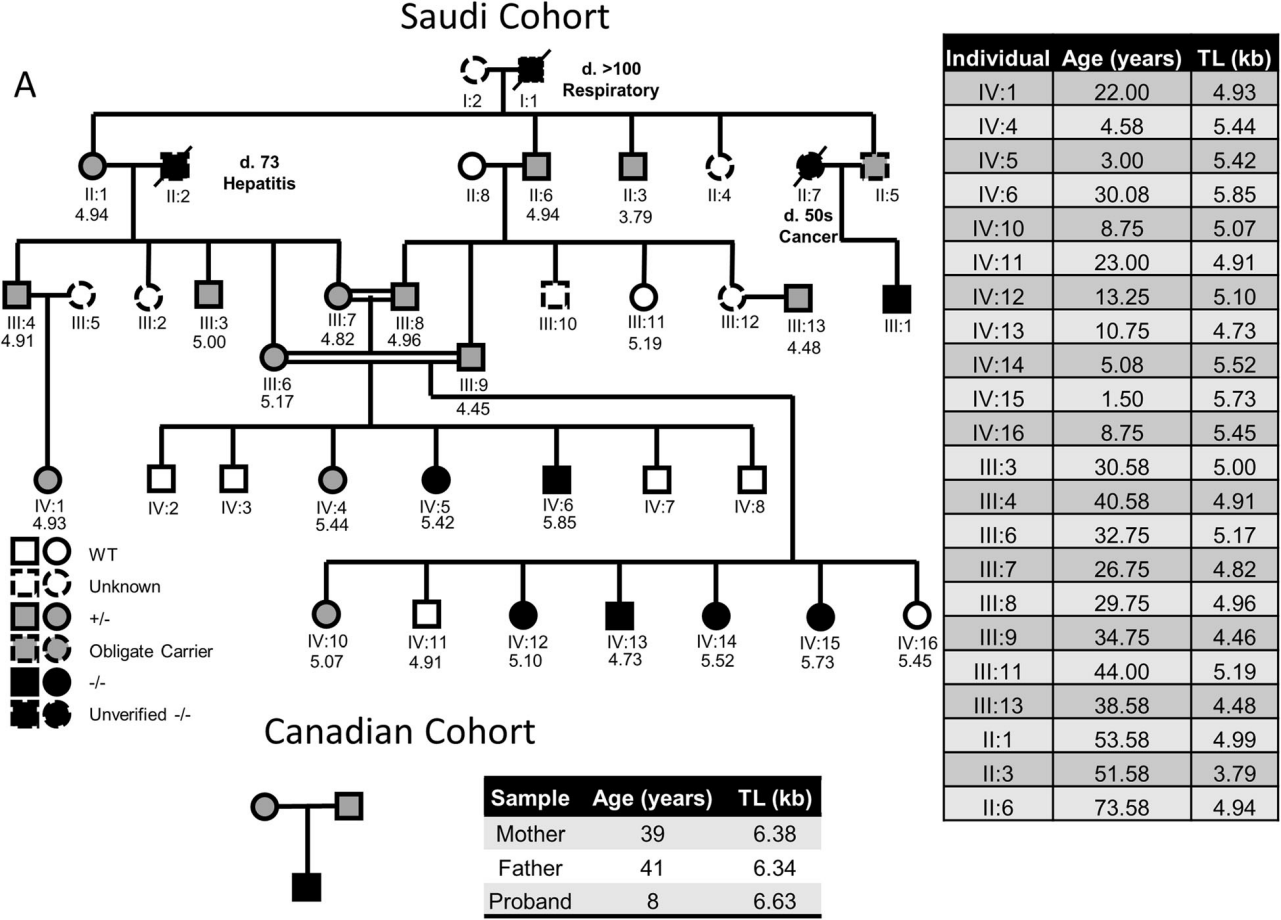
Pedigree of LARP7 mutant cohorts. Progressively shorter initial telomere length in successive generations of the LARP7 mutant Saudi cohort illustrate a pattern of genetic anticipation observed in other telomeropathies. The age (in years) and telomere length (TL) in kilobase pairs (kb) of each individual in this Saudi Arabian pedigree (left side) are provided in the table (right side). The smaller Canadian cohort is detailed at the bottom of this figure (providing age and average telomere length)

Telomeres in affected individuals, carriers and wild-type individuals descended from carriers were all shorter compared to age matched individuals in the general population, as well as compared to an unrelated 28-year-old male control (Fig. 3). Correcting for age-associated telomere shortening with a rate of telomere shortening based on previously conducted longitudinal studies indicates a pattern of anticipation (progressively shorter initial telomere length with each succeeding generation) consistent with observations from other telomeropathies (Fig. 4). Initial telomere length in the Saudi Arabian cohort is decreasing inter-generationally, with a difference of 560 base pairs in initial telomere length between the generation III and generation IV individuals measured, or by roughly 30 base-pairs per year.

**Fig. 5:**
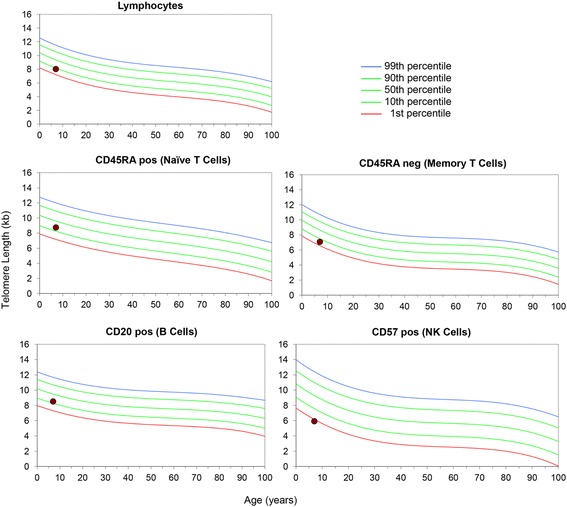
Flow-FISH analysis of telomere length in the Canadian proband. The individual presenting with Alazami syndrome had lymphocyte telomere length below the 10^th^ percentile for his age, and telomere length below the 1^st^ percentile for the CD57+ natural killer cell fraction

Flow-FISH analysis of telomere length from the Canadian proband indicates telomere length in the lower 10^th^ percentile for all leukocytes, with telomere length in CD57 positive cells below the 1^st^ percentile (Fig. 5).

**Fig. 6:**
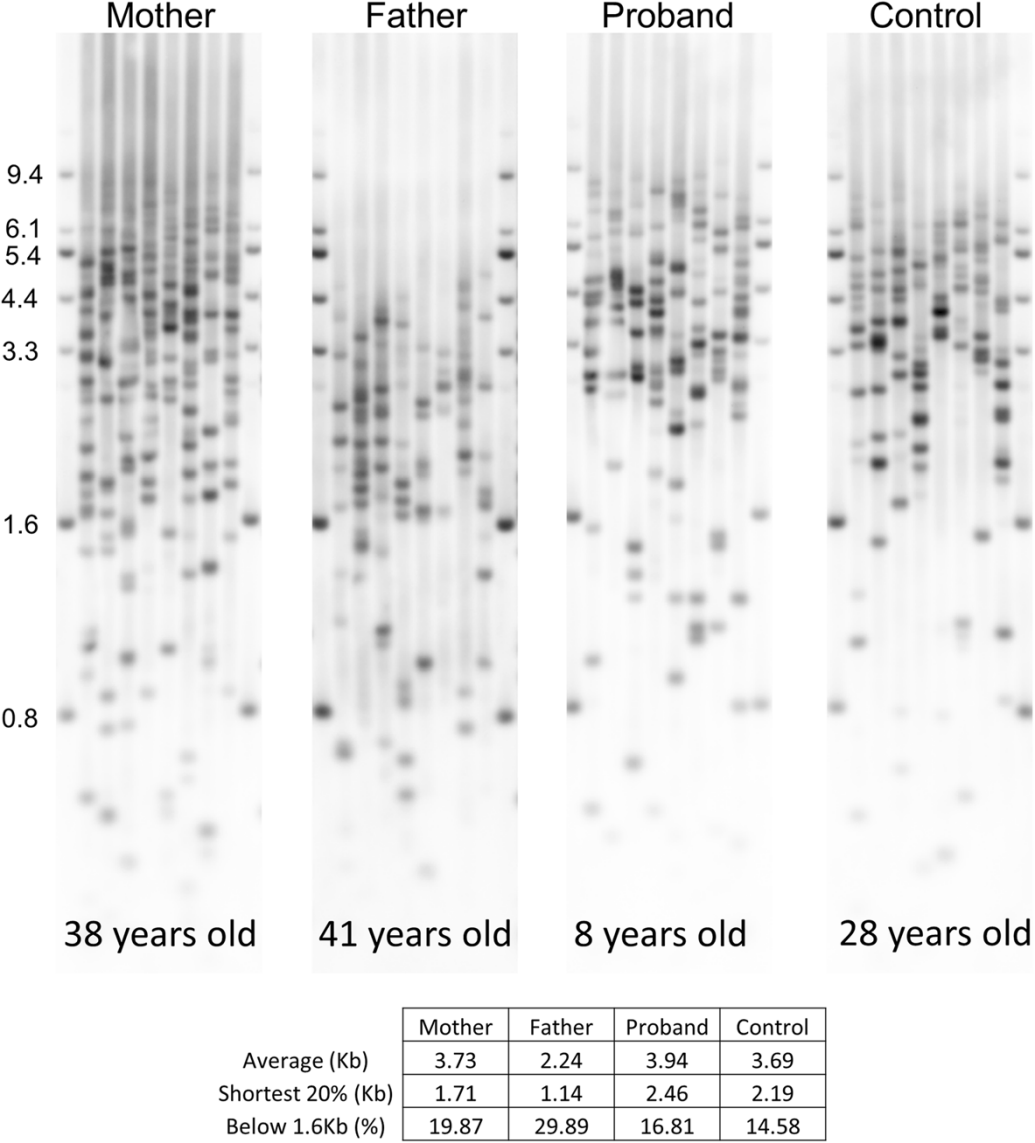
Universal STELA on the Canadian family. The length of the shortest telomeres was reduced in all three members of the Canadian cohort, with Universal STELA products in the 8-year-old proband roughly comparable to an unrelated 28-year-old male

Universal Single Telomere Length Analysis (Uni-STELA) can reveal the length of the shortest telomeres, which matter the most in establishing proliferative limits on the cell [22]. We utilized Uni-STELA to determine if the Canadian cohort had a greater abundance of very short telomeres compared to the control, and to evaluate if there was a difference between the behavior of the bulk telomere population and the shortest telomeres (Fig. 6). Consistent with the TRF observations, the 8 year old Alazami-syndrome affected proband exhibited Uni-STELA telomere lengths roughly equivalent to the 28-year old unrelated control, demonstrating telomere lengths substantially shorter than expected for the age group. The proband’s father also displayed a remarkably short Uni-STELA distribution, with a much greater proportion of Uni-STELA products below 1.6 kb compared to the unrelated control.

## Discussion

At least one other La-domain containing protein associates with telomerase in humans, LARP3 (previously known as simply La) [8], suggesting that evolutionarily conserved activities of *Tetrahymena* p65 may have been allocated to separate La-domain family proteins in humans. This is one possible explanation for the differences between the results of depletion of human LARP7 and *Tetrahymena* p65. It is also possible that the reduction in telomerase enzymatic activity observed is due to the loss of some aspect of the telomerase holoenzyme assembly, processivity or to the loss of full-length TERT mRNA in the LARP7 knockdown cells. The relative reduction in full-length transcript abundance is comparable to the reduction in telomerase enzymatic activity.

The observation that even wild-type individuals in the Saudi Arabian LARP7 cohort have very short telomeres is consistent with what is observed in other telomeropathies, because all wild-type individuals in that cohort are descended from at least two generations of LARP7 mutation carriers. Telomere lengths in those individuals are comparable to wild-type offspring of carriers of nonfunctional TERT alleles [23], suggesting a similarly severe defect in telomere maintenance occurs in the context of LARP7 deficiency. A number of individuals in these families are pre-pubescent, in age ranges wherein telomere shortening is faster than it is in adulthood, and the age-associated shortening rate used to correct for age does not account for this. Thus, it is likely that initial telomere lengths in the most recent generation of the Saudi cohort have not actually stabilized because of this inadequacy in the age correction.

The Canadian family displays a similar pattern of shorter than average telomere length and progressively shorter initial telomere length, albeit to a lesser degree than the Saudi family. Though a number of individuals in the Saudi cohort exhibited microcytic anemia and the Canadian proband exhibited borderline anemia, these could be due to environmental factors such as malnutrition or be otherwise unrelated to the telomere phenotype. Anemia and specific lymphocytopenias are consistent with the symptoms of a telomeropathy [23–25], but the phenotypes observed in these cohorts are not severe enough to say with certainty that they arise from impaired telomere maintenance.

**Fig. 7:**
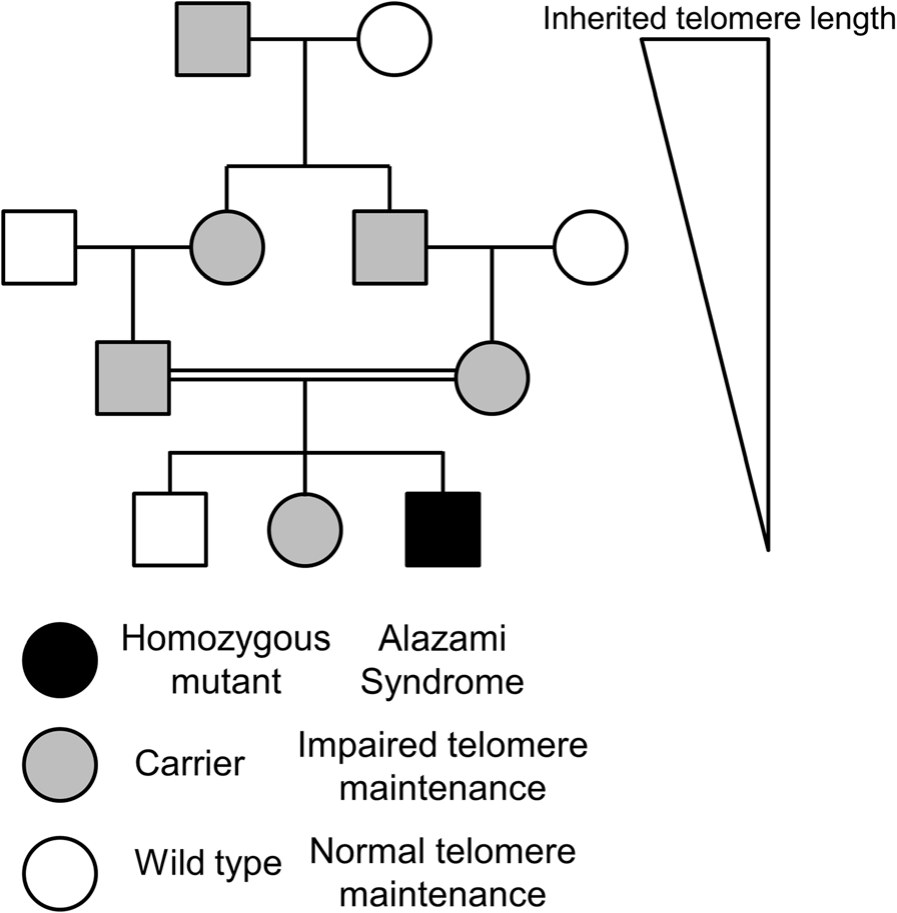
Model of the dominant nature of the impaired telomere maintenance in LARP7 mutant cohorts. In this model, the developmental defects associated with LARP7 deficiency are recessive, whereas the impaired telomere maintenance and inheritance of short telomeres can be transmitted even from heterozygous, overtly normal individuals. Thus the telomere length phenotype of LARP7 deficiency/Alazami syndrome displays a dominant inheritance modality with genetic anticipation in telomere shortening

Because two different cohorts with two distinct loss-of-function mutations in LARP7 display a similar pattern of symptoms as well as impaired telomere maintenance, it is likely that the short telomere phenotype observed here is due to the pathology rather than an inter-ethnic or inter-regional difference in mean telomere length, which has been reported [26]. Because both wild-type and individuals heterozygous for a loss-of-function mutation in LARP7 display short telomeres and progressively shorter initial telomere length, it is reasonable to assume the developmental symptoms of Alazami syndrome are distinct from the telomeric symptoms. We propose a model (Fig. 7) in which an equilibrium telomere length in germ cell progenitors is disrupted even in the context of reduced function of LARP7, haploinsufficiency in the telomeric aspects of LARP7 function, while LARP7’s other functions are sufficiently robust that the developmental phenotypes of Alazami syndrome are recessive in nature.

## Conclusions

We investigated if humans with LARP7 deficiency exhibited symptoms of a telomeropathy or displayed enhanced telomere shortening with time in a previously described cohort of individuals with LARP7 deficiency (Alazami syndrome) [21] as well as another family identified with a mutation in LARP7. We first determined the effects of LARP7 knockdown on telomere length, telomerase (hTERT) splicing and enzymatic activity in human cell culture models, and then assessed the telomere biology of both affected and unaffected relatives of Alazami syndrome patients. We discovered that LARP7 knockdown induces progressive time-dependent telomere shortening concomitant with a reduction in telomerase enzymatic activity and a decrease in full-length (catalytically active) *TERT* mRNA in vitro, and that humans with LARP7 deficiency display dramatically short telomeres, borderline anemia in younger generations, and anticipation consistent with a telomeropathy.

## Abbreviations

CTC1: CTC telomere maintenance complex component 1
ddTRAP: Droplet-digital telomere repeat amplification protocol
DKC1: Dyskeratosis congenita 1
DNA: Deoxyribonucleic acid
FISH: Fluorescent in situ hybridization
hTERT/TERT: Human telomerase reverse transcriptase
LARP3: La-autoantigen related protein 3
LARP7: La-autoantigen related protein 7
mRNA: Messenger ribonucleic acid
NHP2: NHP2 ribonucleoprotein
PARN: Poly(A)-specific ribonuclease
PCR: Polymerase chain reaction
RNA: Ribonucleic acid
RTEL1: Regulator of telomere elongation helicase 1
shRNA: Short hairpin ribonucleic acid
TCAB1: WD repeat containing, antisense to TP53 (alias WRAP53)
TERC: Telomerase RNA component
TIN2/TINF2: TERF1 (TRF1)-interacting nuclear factor 2
TRF: Terminal restriction fragment assay
Uni-STELA: Universal single telomere length analysis
